# Supplementary material for: Surface respiratory electromyography and dyspnea in acute heart failure patients
Source: PLoS One. 2020 Apr 29;15(4):e0232225. doi: 10.1371/journal.pone.0232225 (PMC7190138; doi:10.1371/journal.pone.0232225)
Supplement: Attachment — Submitted filename: review.pdf [file pone.0232225.s001.pdf]

The authors propose to use surface EMG of respiratory muscles as an objective way to detect and monitor short of breath for acute heart failure patients. To this end, they wanted to test the hypothesis that the severity of dyspnea is correlated to an EMG index (mean magnitude normalized by maximum muscle contraction) of respiratory muscles. They designed a procedure and collected data from 25 patients and post processed EMG data. They show that the severity of dyspnea is positively correlated with the magnitude of the EMG and concluded that this could be used as a clinical procedure.

The authors adopted similar approaches studied in the literature. The novelty of the paper is in its application with HF patients. The organization of the paper is good. The reviewer has a few comments as following:

(1) Could the authors include the total time needed for the procedure? Including sensor placement, skin preparation, etc.

(2) The interpretation of table 2 is not clear. The authors included many clinical measurements without explaining them.

(3) Speaking of table 2, it seems the respiratory rate would correlate well with both Likert and EMG. So why not use this variable to indicate short of breath instead of using EMG? After all, as the authors mention, EMG has so many limitations in practice.

(4) One of the disadvantages of surface EMG that the authors did not mention is that it registers heartbeat, as shown in figure 1. Did authors apply filters to remove/minimize this effect? Was this the reason why the authors placed all electrodes on the right side of the body?

(5) The statistical analysis is not complete. For linear correlation, please report the method used, normality of variables, residuals, normality of residuals, etc. In addition to P value, please also report F value.

(6) It's unclear how the technology will be used in clinic. One of the advantages to use EMG over Likert, according to the authors, is that it doesn't require patient cooperation. This is a little bit confusing because the procedure requires the patient to breathe at maximum effort for several cycles.
